# Structured Vs Conventional Endoscopic Vein Harvesting Training: Vein Integrity and Learning Curve Performance

**DOI:** 10.3310/nihropenres.14474.1

**Published:** 2026-10-23

**Authors:** Bhuvaneswari Krishnamoorthy, Sam Raaj, Rick Air, Cristina Ruiz Segria, Raquel Enriquez, Setemi Olufemi, Jaydeep Sarma, James Barnard, Azita Rajai, Enoch Akowuah, Rajamiyer Venkateswaran

**Affiliations:** 1 Directorate of Nursing and Midwifery, University of Salford School of Health and Society, Salford, England, UK; 2 Department of Cardiac surgery, Manchester University NHS Foundation Trust, Manchester, England, UK; 3 Department of Cardiovascular sciences, Faculty of Health & Society, The University of Manchester, Manchester, England, UK; 4 Faculty of Medicine, Medical School, Imperial College London, London, England, UK; 5 Department of Cardiac surgery, South Tees Hospitals NHS Foundation Trust, Middlesbrough, England, UK; 6 Centre for Biostatistics, Manchester Academic Health Science Centre, The University of Manchester, Manchester, England, UK; 7 Translational and Clinical research Institute and Academic Cardiovascular Unit, Newcastle University Faculty of Medical Sciences, Newcastle upon Tyne, England, UK

**Keywords:** Endoscopic vein harvesting, Coronary artery bypass surgery, Structured training, vein endothelial integrity, CT angiogram, immunohistochemistry, Beck Anxiety Inventory, Global Operative Assessment of Laparoscopic skills.

## Abstract

**Background:**

Endoscopic Vein Harvesting (EVH) reduces wound complications compared with open vein harvesting. However, concerns remain that conduit injury may occur during the learning curve . The impact of EVH training on vein integrity and progression to independent practice remains unclear . We evaluated whether a structured EVH training programme improved vein quality, technical skill and trainee confidence.

**Methods:**

This study was originally designed as a multicentre cluster-randomised trial but was prematurely halted and completed as a two-centre study with cluster allocation. Four EVH trainees were included: Two received Structured Training (ST) incorporating simulation, psychomotor reinforcement, video review and competency-based progression. Two underwent Conventional Training (CT), consisting of standard company-led training.

Endothelial integrity was assessed using CD34 immunohistochemistry across proximal, random and distal sections. Endothelial stretching and muscle hypertrophy were graded. Trainee performance, anxiety and consultant-rated vein quality were recorded. Patients were followed to 12 months for Major Adverse Cardiac Events and 24 months for survival. Analyses were descriptive.

**Results:**

Forty-six patients underwent EVH (ST n = 40; CT n = 6). Technical performance improved in all trainees but CT trainees discontinued independent EVH practice during the study period. ST trainees demonstrated better endothelial integrity with less stretching and muscle hypertrophy. CT vein samples showed greater endothelial variability.

Clinical event rates were low. At 6–24 months, MACE was infrequent with repeat angina (4.3%), myocardial infarction (2.2%), re-intervention (6.5%) and non-cardiac death (2.2%). Among the 38 vein grafts assessed by computed tomography angiography in asymptomatic patients, 23 (60.5%) were patent, five (13.2%) demonstrated flow-limiting stenosis, and 10 (26.3%) were occluded. These findings were predominantly observed in small-calibre target vessels in patients with diabetes.

**Conclusion:**

Structured EVH training was associated with better endothelial preservation during the learning curve. Given the small sample size and incomplete imaging follow-up, these findings are hypothesis-generating and require validation in larger multicentre studies.

**Clinical registration:** ISRCTN27493194; February 2022; amended 2024.

This work has been presented at International Society of Minimally Invasive Surgery (ISMICS) annual conference meeting as a e-poster online on 10-13 ^th^ June 2026. e-poster online; Available at ismics2026.109014b.NORMAL.pdf

## Plain Language summary

**Why was this study done?:**

Endoscopic Vein Harvesting (EVH) is a keyhole surgery used to remove leg veins for coronary surgery. Compared with the traditional open surgery, EVH reduces wound pain, infection and scarring. However, concerns remain about whether EVH can be learned safely and whether the blood vessel quality may be affected while trainees are developing their skills. We wanted to explore whether a structured training could improve technical skills and help preserve vein quality compared with company training.

**What did we do?:**

We studied four trainees learning EVH. Two trainees completed a structured training programme that included simulation practice, video feedback, psychomotor skills training and competency-based progression. Two trainees received conventional company-led training. We examined the quality of harvested veins under a microscope, assessed trainee performance and confidence and followed patients.

**What did we find?:**

A total of 46 patients underwent EVH. All trainees improved their technical skills over time. However, the two trainees who received standard training stopped performing EVH independently during the study, whereas those who completed the structured programme continued to perform.

Serious heart-related complications were uncommon during follow-up. Between 6 and 24 months after surgery, 4.3% of patients experienced recurring chest pain, 2.2% had a heart attack, 6.5% required a further procedure and 2.2% died from causes unrelated to their heart condition. In a small group of patients who underwent a special heart scan, most blood vessel grafts remained open. However, some grafts showed reduced blood flow or were blocked, particularly in smaller blood vessels and in patients with diabetes.

**What do these findings mean?:**

This small study suggests that structured EVH training may help trainees develop and maintain their skills while preserving vessel quality during the learning process. Larger studies are needed to confirm whether structured training leads to better long-term patient results.

## Introduction

Although the internal mammary artery is the gold standard conduit for Coronary Artery Bypass Grafting (CABG). The Long Saphenous Vein (LSV) remains the most frequently used conduit for multivessel revascularisation due to its accessibility and length. ^1^ Open Vein Harvesting (OVH) requires a longitudinal incision up to 90 cm and is associated with pain, delayed wound healing and reduced patient satisfaction. ^2^ Since its introduction in 1996, Endoscopic Vein Harvesting (EVH) performed through a 2-3 cm incision has reduced morbidity and improved patient-reported outcomes. ^2^ ^,^ ^3^

Studies report no differences in Major Adverse Cardiac Events (MACE) between OVH and EVH. ^4^ ^,^ ^5^ ^,^ ^6^ However, EVH has a recognised learning curve during which conduit injury may occur. Concerns regarding graft quality relate to anatomical variability and commercially led training models. ^7^ ^,^ ^8^ ^,^ ^9^ Structured standardised training may improve technical proficiency and conduit preservation. ^9^ While EVH offers clear benefits in reducing wound morbidity, conduit quality is influenced by operator experience with improvements observed as proficiency develops. This study was completed as a two-centre study with cluster allocation to conventional training (CT) or structured training (ST) without any individual randomisation due to COVID-19 pandemic.

## Methods

Ethical approval was obtained from Northwest - Greater0 Manchester South Research Ethics Committee (REC reference: 22/NW/0010) and was prospectively registered (ISRCTN27493194; February 2022; amended 2024). The study was originally designed as a multicentre cluster-randomised trial (CREST) across 6 hospitals (12 trainees, 240 patients) but was prematurely halted due to the COVID-19 pandemic and NHS service pressures. It was completed in two hospitals (Manchester Foundation NHS Trust (MFT) and St. James Cook University hospital in South Teesside (STH)) involving four trainees and 46 patients. Written informed consent was obtained from trainees and patients between March 2022 and March 2025. ^10^ Patients were prospectively recruited post-training but not strictly consecutive due to trainee availability and service constraints.

### Techniques and intervention

Four Surgical Care Practitioners had comparable prior experience (>250 OVH cases) but no EVH exposure. Variability in baseline knowledge and performance was observed and equivalence between trainees cannot be assumed. All CABG procedures were performed by consultant cardiac surgeons with varying levels of experience. Emergency and off-pump CABG surgery patients were excluded. Trainees with <12 months post-qualification experience, prior EVH experience, lack of smartphone access or unwillingness to participate in mobile games were excluded.

### Structured training: (ST - MELT group)

Two trainees (T1 & T2) were recruited eight weeks before EVH training and completed four-step MELT training. ^7^

- **Step 1** comprised knowledge-based training (2 weeks).
- **Step 2** focused on skill development including ultrasound vein mapping, leg-model simulation practice, and mobile gaming (Temple Run) for hand–eye coordination (4 weeks).
- **Step 3** involved practical exposure including supervised observation, review of EVH training videos and familiarisation with EVH equipment (2 weeks).
- **Step 4** comprised a staged progression of EVH training over 11 weeks, with trainees initially harvesting a single vein segment, followed by two- and three-segment harvests, before progressing to harvesting in increasingly complex patients.

### Conventional training: (CT group)

Two trainees (T3 & T4) underwent conventional EVH training according to the Getinge™ company training protocol. To minimise bias, the company trainer was not provided with the study protocol. According to principal investigator records, both trainees completed the company e-learning platform approximately three weeks before EVH training and undertook limited leg-model simulation to familiarise themselves with EVH equipment. Trainees then progressed to supervised clinical cases with a company proctor present for an initial 2-week continuous training period, after which independent practice was expected.

### Both groups

All procedures used the Maquet VasoView Hemopro 2 system with intraoperative heparinisation. ^3^ ^,^ ^11^ In the ST trainee group, progression to independent harvesting required ≥80% competency as assessed by the trainer using MELT. In the CT trainee group, progression was determined by the company trainer according to company protocol without a formalised competency threshold.

Vein quality was assessed using structured scoring protocols. ^3^ CD34 immunohistochemistry quantified endothelial integrity (0–100%). ^4^ ^,^ ^12^ Smooth muscle damage (Masson’s Trichrome) and endothelial stretching/detachment (H&E) were graded (0–4 scale). Sections were independently scored by three blinded assessors and validated by a consultant histopathologist. Formal inter- and intra-observer variability analysis was not performed.

Trainee outcomes were evaluated using the Kirkpatrick framework ^13^ (reaction, learning, behaviour, results). Technical performance was assessed using the Global Operative Assessment of Laparoscopic Skills (GOALS; 25-point validated scale), ^14^ and anxiety was measured using the Beck Anxiety Inventory ^15^ (BAI). Assessments were conducted pre-, during and post-training. “During training” refers to the supervised phase of EVH skill acquisition. “Post training” refers to performance following progression to independent practice or near-independent practice. Outcome assessors and data analyst were blinded.

### Study endpoints

 The primary outcome was histological conduit damage. Additional endpoints included the incidence of MACE and quality of life measured using EQ-5D-5L. MACE comprised repeat angina, dyspnoea, myocardial ischemia/infarction, reintervention, stroke and death and was determined through telephone interviews, outpatient records, general practitioner letters and coroner reports at 3-month intervals up to 12 months postoperatively.

### Statistical analysis

Due to the absence of patient-related samples from trainees at STH hospital, the analysis was mainly descriptive and no comparative analysis between ST and CT trainees was performed. Data were summarised as frequencies/percentages for categorical variables and as median and interquartile range [IQR] for continuous variables. Endothelial integrity (CD34 expression) is presented as median percentage while other histological outcomes are presented as median scores.

## Results

### Demographics

Four trainees and 57 patients provided written informed consent. Eleven patients (19%) were excluded due to surgery cancellation and performed by different operators ( Figure 1). EVH was performed by four trainees in 46 patients: 40 at MFT (ST group, T1 & T2) and six patients at STH hospital (CT group, T3 & T4). ( Table 1). Trainees within each group underwent the same training pathway within their respective centres. The observed differences between individual trainees likely reflect variation in learning and case exposure rather than systematic differences. ST trainees continued EVH practice (>400 cases each) whereas CT trainees discontinued EVH practice in June 2024. Histological evaluation included 138 veins (406 analysable sections after 8 exclusions) with proximal (H1), random/distended (H2) and distal (H3) sections ( Figure 1).

**Figure 1.**
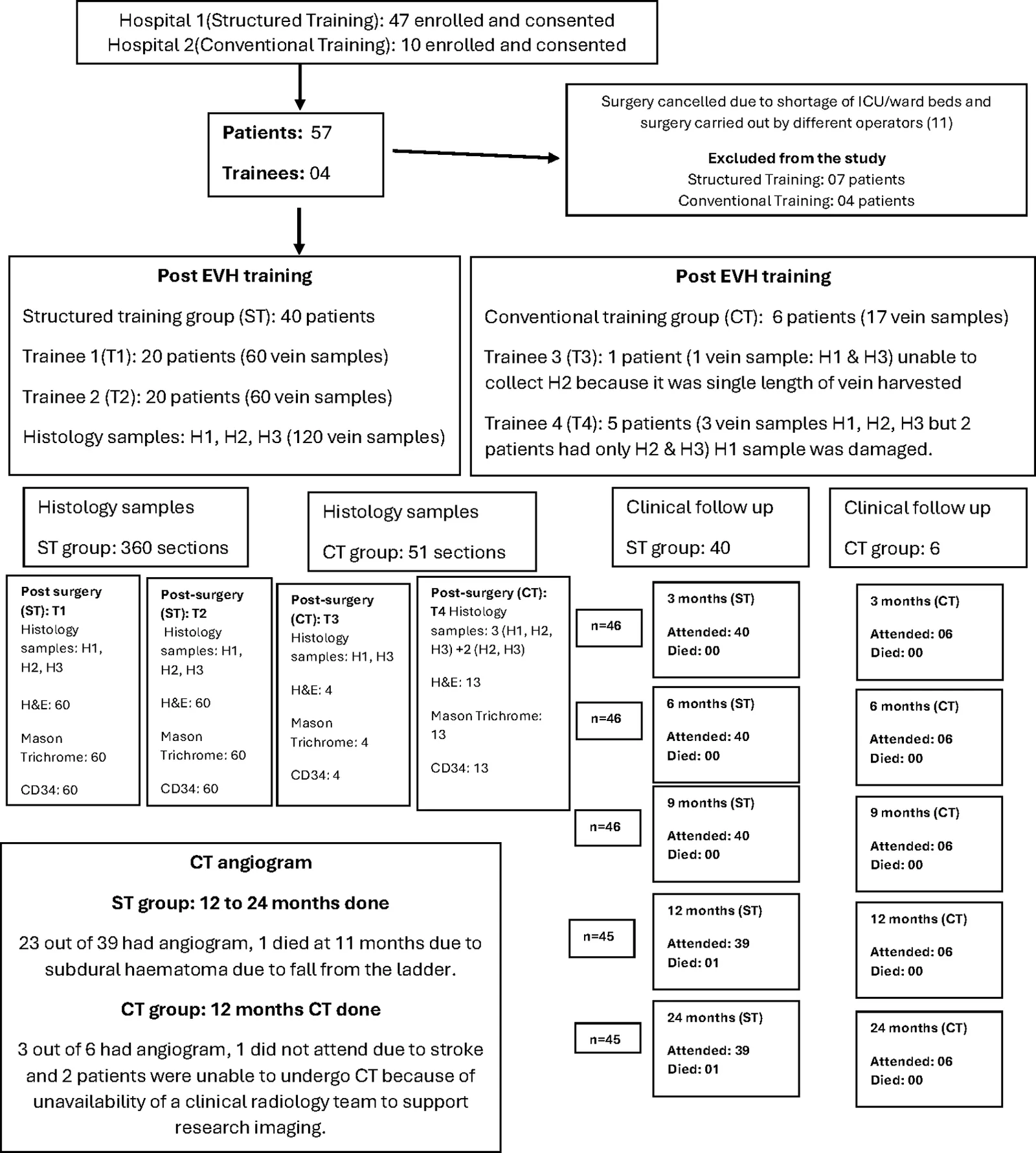
Consort patient and trainee flow diagram.

**Table 1.** Demographics data including preoperative comorbidities, risk factors and intraoperative variables.

Demographic variables	T1 (ST, n = 20)	T2 (ST, n = 20)	T3 (CT, n = 01)	T4 (CT, n = 05)
Age	62.0 [54.5, 68.0]	68.5 [59.0, 76.0]	NA	68.0 [56.0, 76.0]
M/F, n(%)	17.0 /3.0 (85.0/15.0)	17.0 /3.0 (85.0/15.0)	1.0/0.0	5.0/0.0
Body mass index Median [IQR]	28.5 [25.7, 31.6]	26.9 [25.4, 30.5]	30.0 [30.0, 30.0]	29.0 [28.0, 29.0]
**Surgery, n(%)**				
Elective	8.0 (40.0)	6.0 (30.0)	0.0 (0.0)	3.0 (60.0)
Urgent-in-patient	12.0 (60.0)	14.0 (70.0)	1.0 (100.0)	2.0 (40.0)
**Diabetes mellitus, n(%)**				
Diet controlled	9.0 (45.0)	12.0 (60.0)	0.0	2.0 (40.0)
Tablet controlled	6.0 (30.0)	8.0 (40.0)	0.0	0.0
Insulin controlled	4.0 (20.0)	2.0 (10.0)	0.0	0.0
**Smoking, n(%)**				
Never smoked	9.0 (45.0)	9.0 (45.0)	0.0	2.0 (40.0)
Previous smoker	7.0 (35.0)	8.0 (40.0)	1.0 (100.0)	2.0 (40.0)
Current smoker	4.0 (20.0)	3.0 (15.0)	0.0	1.0(20)
**Left ventricular ejection fraction, n(%)**				
>50%	13.0 (65.0)	16.0 (80.0)	1.0 (100.0)	3.0 (60.0)
30%–50%	6.0 (30.0)	4.0 (20.0)	0.0 (0.0)	2.0 (40.0)
<30%	1.0 (5.0)	0.0 (0.0)	0.0 (0.0)	0.0 (0.0)
Logistic Euroscore				
Median [IQR]	2.5 (1.6, 3.1)	3.4 (2.2, 5.7)	NA	NA
Missing data	0	0	1	5
STEMI n(%)	5.0 (25.0)	2.0 (10.0)	0.0 (0.0)	0.0 (0.0)
NSTEMI n(%)	7.0 (35.0)	11.0 (55.0)	1.0 (100.0)	5.0 (100.0)
Previous PTCA, n(%)	5.0 (26.3)	4.0 (20.0)	0.0 (0.0)	0.0 (0.0)
Presence of Q wave	13.0 (65.0)	5.0 (25.0)	0.0 (0.0)	0.0 (0.0)
Multivessel coronary disease, n(%)	19.0 (95.0)	20.0 (100.0)	1.0 (100.0)	5.0 (100.0)
Left main stem disease, n(%)	6.0 (30.0)	3.0 (15.0)	1.0 (100.0)	2.0 (40.0)
Hypercholesterolemia n(%)	19.0 (95.0)	13.0 (65.0)	1.0 (100.0)	4.0 (80.0)
Hypertension, n(%)	16.0 (80.0)	17.0 (85.0)	1.0 (100.0)	5.0 (100.0)
Peripheral vascular disease, n(%)	1.0 (5.0)	0.0 (0.0)	0.0 (0.0)	0.0 (0.0)
**Intraoperative variables**				
Number of grafts Median [IQR]	3.0 [2.0, 4.0]	3.0 [2.0, 4.0]	2.0 [2.0, 2.0]	3.0 [2.0, 3.0]
Number of vein grafts Median [IQR]	2.0 [1.0, 2.5]	2.0 [1.5, 3.0]	1.0 [1.0, 1.0]	2.0 [1.0, 2.0]
Vein graft not used for surgery, n(%)	2.0 (10.0)	0.0 (0.0)	0.0 (0.0)	0.0 (0.0)
Leg drain inserted, n(%)	18.0 (94.7)	20.0 (100.0)	0.0 (0.0)	0.0 (0.0)
Bypass time, Median [IQR]	101.0 [84.0, 137.0]	99.0 [83.0, 127.0]	51.0 [51.0, 51.0]	78.0 [69.0, 108.0]
X Clamp time, Median [IQR]	62.0 [53.0, 86.0]	67.0 [52.0, 85.0]	30.0 [30.0, 30.0]	66.0 [49.0, 93.0]
Vein harvesting time (min)	55.0 (48.5, 67.0)	54.5 (43.0, 68.0)	NA	65.0 (60.0, 66.0)
Total surgery time (min)	245.0 (221.3, 278.5)	260.0 (202.5, 292.5)	NA	220.0 (205.0, 246.0)
Leg surgery (min)	98.0 (76.0, 114.5)	94.0 (78.1, 06.5)	NA	71.0 (65.0, 90.0)
2500 IU systemic Heparin given before starting EVH	16.0 (80.0)	19.0 (95.0)	1.0 (100.0)	5.0 (100.0)

CCS: Canadian Cardiovascular Society; PTCA: Percutaneous Coronary Angioplasty.

### Endothelial integrity: CD34

Across all sections (H1-H3), the ST group demonstrated consistently high median endothelial integrity, generally above 90% in proximal (H1) and distal (H3) and slightly lower values in random sections (H2). Median endothelial integrity was lower in the CT group. ( Figure 2a; Figure 3). Table 2 provides detailed median scores for CD34, H&E and MT staining.

**Figure 2.**
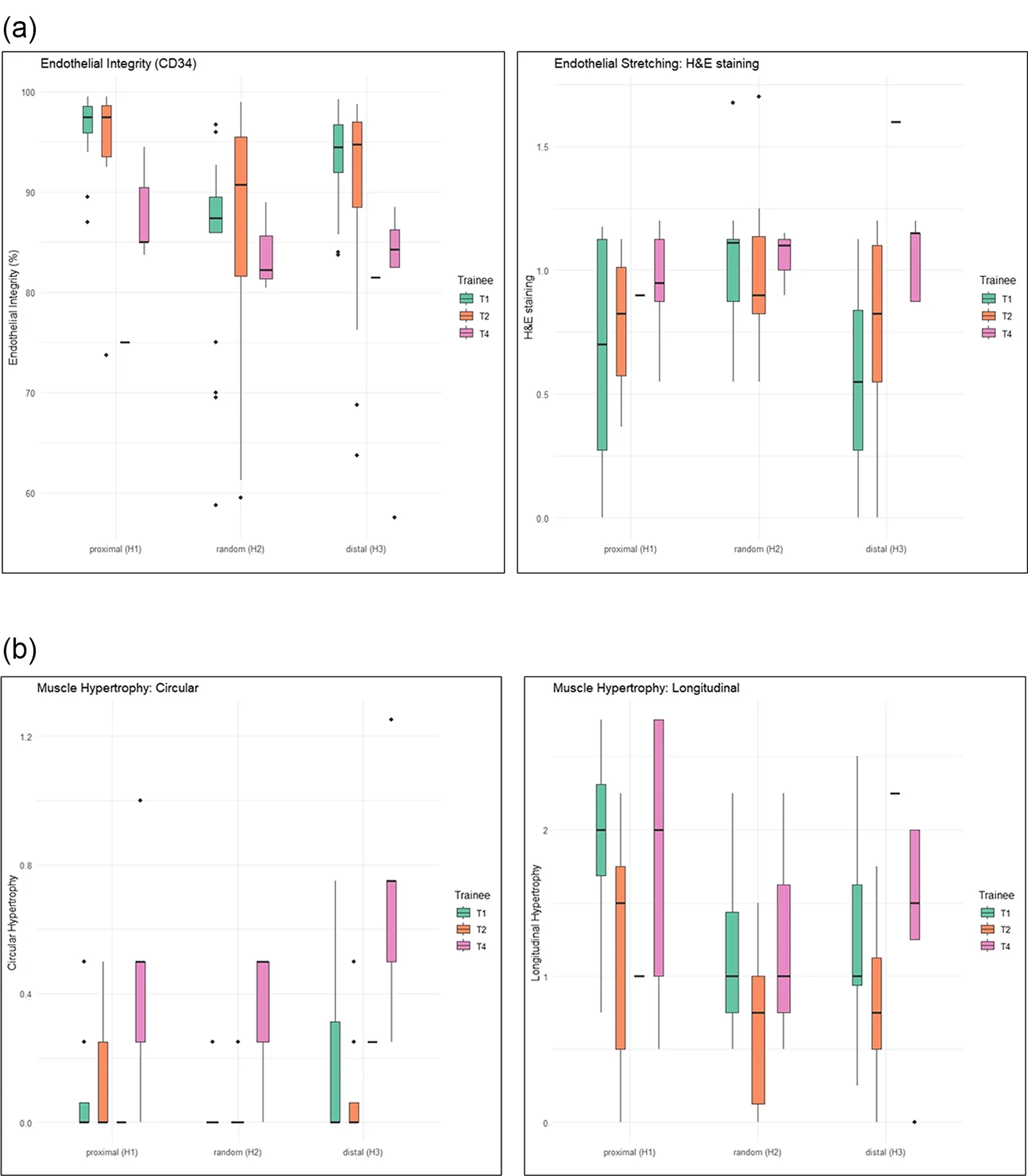
a: illustration of the median percentage of endothelial integrity and stretching on proximal, random and distal vein samples. Figure 2 b: Illustration of the median percentage of muscular hypertrophy of circular and longitudinal muscle on proximal, random and distal vein samples.

**Figure 3.**
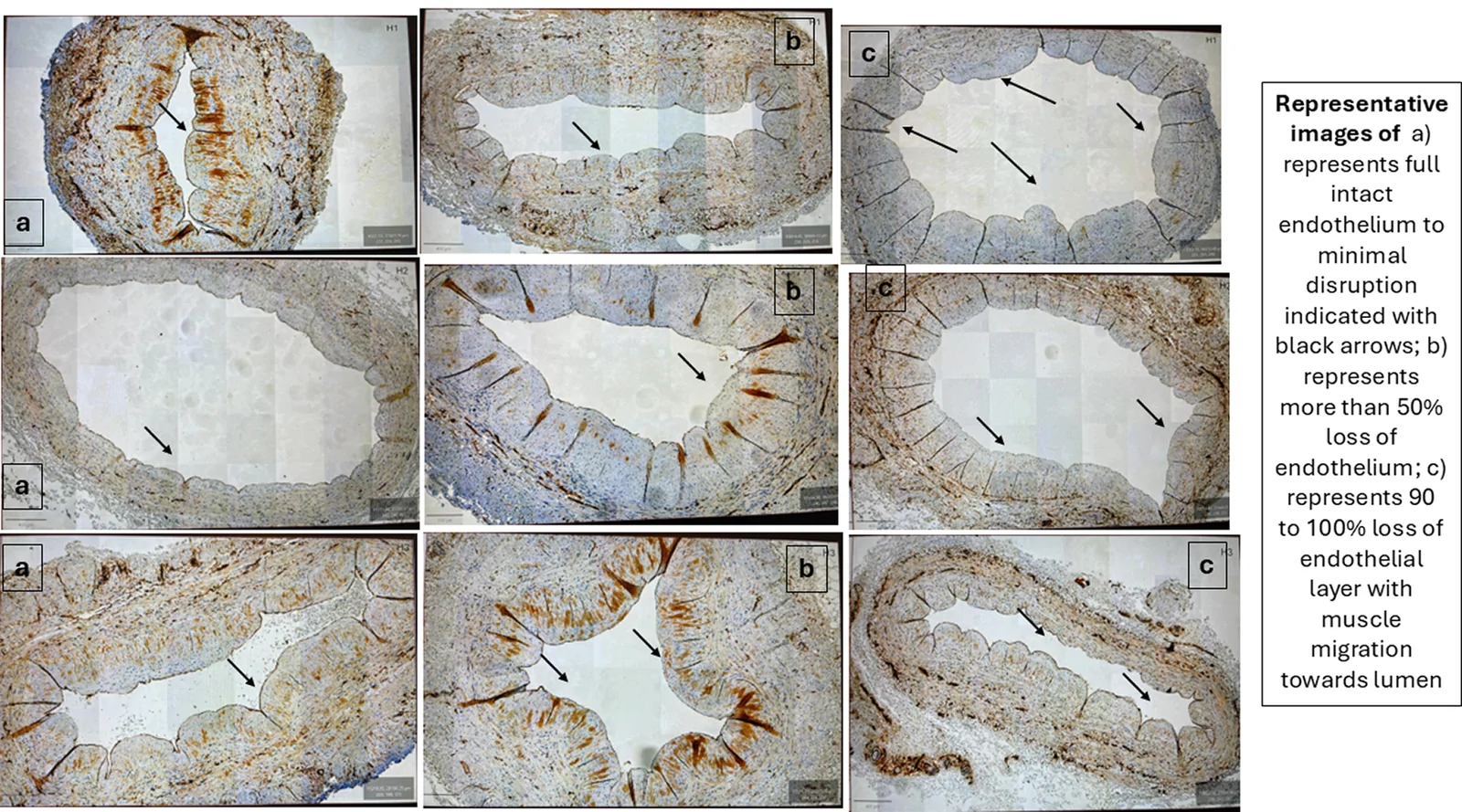
Illustrates the CD34 staining of H1, H2, H3 sections. a) represents full intact endothelium to minimal disruption indicated with black arrows; b) represents more than 50% loss of endothelium; c) represents 90 to 100% loss of endothelial layer with muscle migration towards lumen.

**Table 2.** illustrates endothelial integrity (CD34 staining), Masson’s trichrome staining and Haematoxylin and Eosin (H&E) staining.

Endothelial Integrity (CD34 staining)				
	T1(n = 20)	T2 (n = 20)	T3 (n = 1)	T4 (n = 5)
**H1 sample**				
Median [IQR]	97.5 [95.9, 98.6]	97.5 [93.5, 98.8]	75.0	85.0 [85.0, 90.5]
Missing sample	0	1	0	0
**H2 sample**				
Median [IQR]	87.4 [86.0, 89.8]	90.8 [79.5, 95.5]	Not Available (NA)	82.3 [80.5, 89.0]
Missing	2	1	1	2
**H3 sample**				
Median [IQR]	94.5 [90.8, 97.0]	94.8 [87.3, 97.3]	81.5	84.3 [82.5, 86.3]
Missing	1	0	0	0
**Endothelial stretching (H&E staining)**				
**H1 sample**				
Median [IQR]	0.7 (0.3, 1.1)	0.8 (0.6, 1.1)	0.9	1.0 (0.9, 1.1)
Missing sample	0	1	0	0
**H2 sample**				
Median [IQR]	1.1 (0.9, 1.1)	0.9 (0.8, 1.2)	NA	1.1 (0.9, 1.2)
Missing	2	1	1	2
**H3 sample**				
Median [IQR]	0.6 (0.3, 0.9)	0.8 (0.6, 1.1)	1.6	1.2 (0.9, 1.2)
Missing	1	0	0	0
**Smooth muscle hypertrophy (Mason Trichrome staining**				
**H1 Longitudinal hypertrophy (LH)**				
Median [IQR]	2.0 (1.6, 2.4)	1.5 (0.5, 1.8)	1.0	2.0 (1.0, 2.8)
Missing sample	0	1	0	0
**H2 (LH)**				
Median [IQR]	1.0 (0.8, 1.5)	0.8 (0.0, 1.0)	NA	1.0 (0.5, 2.3)
Missing sample	2	1	1	2
**H3 (LH)**				
Median [IQR]	1.0 (0.9, 1.8)	0.8 (0.5, 1.3)	2.3	1.5 (1.3, 2.0)
Missing sample	1	0	0	0
**H1 Circular hypertrophy (CH)**				
Median [IQR]	0.0 (0.0, 0.1)	0.0 (0.0, 0.3)	0.0	0.5 (0.3, 0.5)
Missing sample	0	1	0	0
**H2 (CH)**				
Median [IQR]	0.0 (0.0, 0.0)	0.0 (0.0, 0.0)	NA	0.5 (0.0, 0.5)
Missing sample	2	0	1	2
**H3 (CH)**				
Median [IQR]	0.0 (0.0, 0.4)	0.0 (0.0, 0.1)	0.3	0.8 (0.5, 0.8)
Missing sample	1	0	0	0

### Endothelial stretching: H&E

Endothelial stretching was low across all sections in the ST group (median ≤ 1.1). In contrast, the CT group demonstrated numerically higher stretching values, particularly in distal sections (H3, median ≥ 1.2). ( Figure 4).

**Figure 4.**
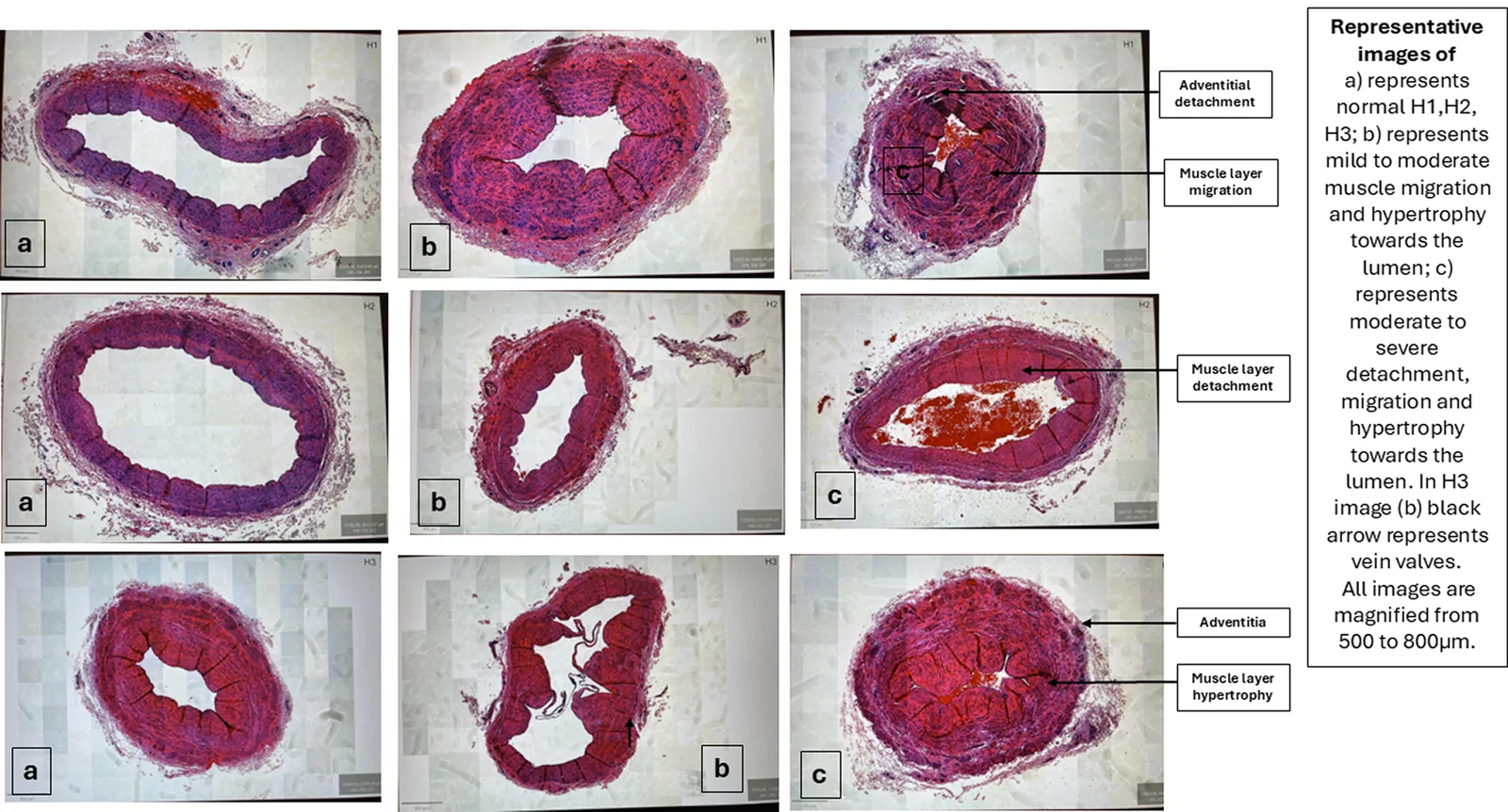
Illustrates the H&E staining of proximal (H1), random (H2) and Distal (H3). All images are magnified from 500 to 800 μm. a) represents normal H1, H2, H3; b) represents mild to moderate muscle migration and hypertrophy towards the lumen; c) represents moderate to severe detachment, migration and hypertrophy towards the lumen. In H3 image (b) black arrow represents vein valves.

### Muscle hypertrophy: masson trichrome

Muscle hypertrophy was consistently low in the ST group across all vein sections ( Figure 5) indicating preserved morphology. Longitudinal hypertrophy in the ST group was mild (median 0.8–2.0) with lower scores in T2. Circular hypertrophy was minimal in the ST group across all sections (median values predominantly 0.0). In contrast, CT group demonstrated greater variability and higher circular hypertrophy, particularly in distal samples (median 0.3–0.8) ( Figure 2b).

**Figure 5.**
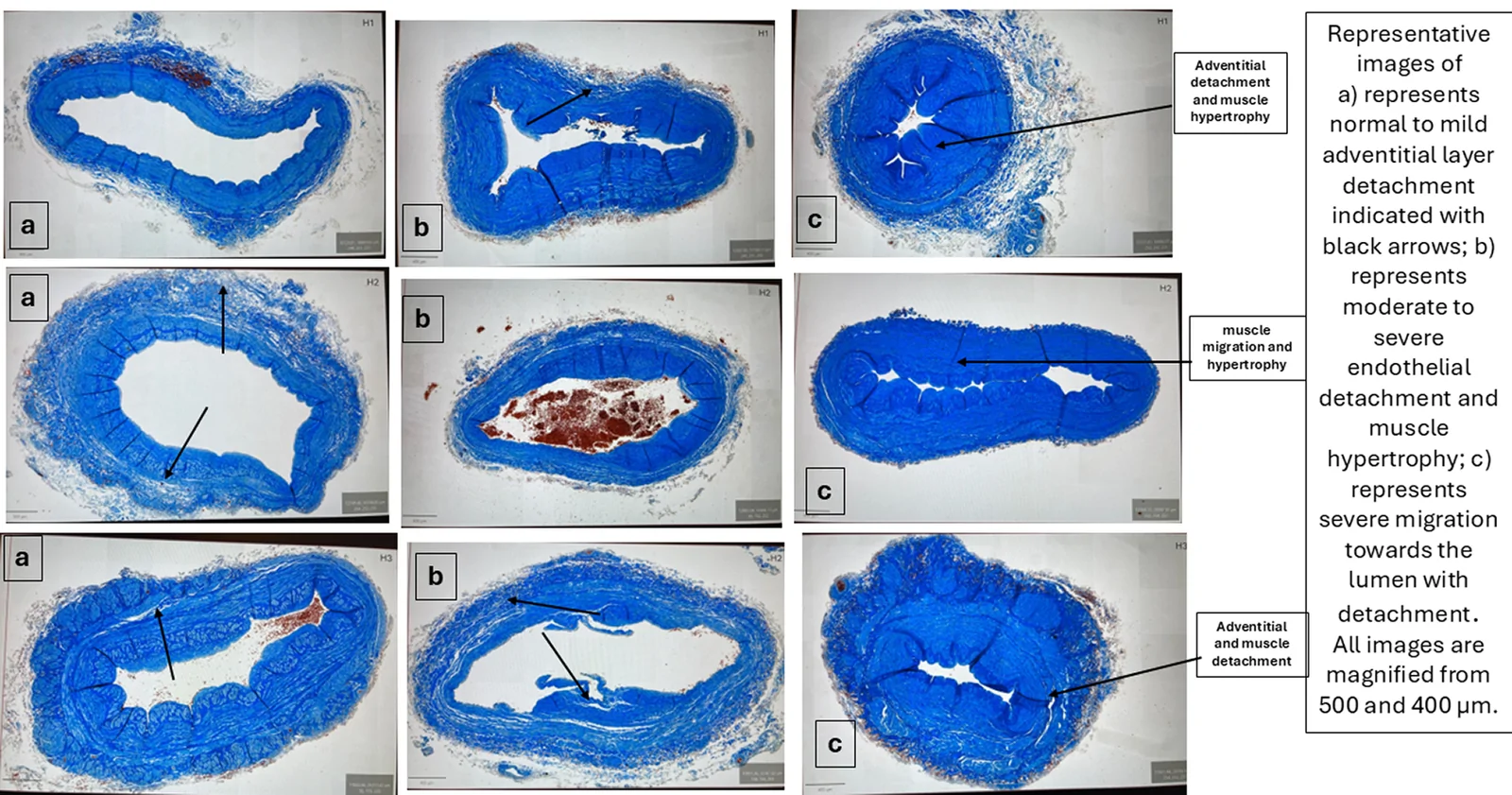
Illustrates the Masson Trichrome staining of H1, H2, H3 sections. All images are magnified from 500 and 400 μm. a) represents normal to mild adventitial layer detachment indicated with black arrows; b) represents moderate to severe endothelial detachment and muscle hypertrophy; c) represents severe migration towards the lumen with detachment.

### Trainee outcomes: pre EVH training tests

**Conventional Training:**

Baseline Single Best Answer (SBA) of LSV anatomy & physiology (8 questions) and EVH procedural knowledge (2 questions) demonstrated low pre-training scores in CT group (T3: 30%; T4: 20%), with most incorrect responses attributed to knowledge recall deficits. Post-training SBA scores showed modest improvement (T3: 40%; T4: 45%). CT trainees subsequently discontinued EVH practice in June 2024.

**Structured training:**

In ST group, SBA pre-training assessments of LSV anatomy & physiology (8 questions) and EVH procedural knowledge (2 questions) yielded scores of 36% (T1) and 20% (T2). Trainees then completed an online e-learning covering CABG, vessel preservation, EVH procedure and molecular mechanisms followed by post-training examination achieving scores of 80% (T1) and 63% (T2).

During step 2, trainees played Temple Run 2™ (Imangi studios, North Carolina, USA) for a minimum of five sessions daily over 14 days. Mean (±SD) performance metrics were: T1 (minutes played 25.42 ± 6.2, sessions/day 8.3 ± 3.4, highest daily score 336,992 ± 214,517) and T2 (minutes played 25.79 ± 7.5, sessions/day 9.6 ± 3.9, highest daily score 236,568 ± 108,578). Trainees were encouraged to continue gaming thereafter to reinforce hand-eye coordination and cognitive responsiveness. ^7^

**Additional outcomes: trainee**

Median GOALS scores improved for all trainees from training to post-training, indicating increased technical competence. During training, median scores ranged from 15.0–21.0, rising post-training to 23.0–25.0 across all trainees. Median vein quality scores were generally good across trainees (median 0–2), improving slightly post-training (1–2). Median BAI scores were low across all trainees during and after EVH training, indicating minimal self-reported anxiety ( Table 3).

**Table 3.** illustrates trainee performance using the Global Operative Assessment of Laparoscopic Skills (GOALS), vein quality, Total Beck Anxiety Inventory (BAI) and Kirkpatrick evaluation outcomes.

Measurement	Structured Training		Conventional Training	
	During training n = 36	Post training n = 40	During training n = 39	Post training n = 8
**Total GOAL score (Scored by independent expert assessor)**				
Median [IQR]	21.0 [15.0, 23.0]	23.0 [23.0, 23.0]	15.0 [15.0, 25.0]	25.0 [25.0, 25.0]
Mean (SD)	18.7 (5.2)	22.9 (1.5)	18.7 (5.1)	25.0 (0.0)
Missing	3	0	0	1
**Total quality of harvested vein score (Consultant surgeon score)**				
Median [IQR]	2.0 [1.0, 2.0]	1.0 [1.0, 3.0]	0.0 [0.0, 2.0]	2.0 [2.0, 2.0]
Mean (SD)	2.0 (1.3)	1.9 (1.6)	1.1 (1.4)	2.0 (1.3)
Missing	3	0	0	2
**Total Beck Anxiety Inventory Score (Self-reported)**				
Median (IQR)	1.0 [0.0, 3.0]	1.0 [0.0, 1.0]	1.0 [0.0, 3.5]	1.0 [0.3, 1.0]
Mean (SD)	1.9 (2.6)	0.8 (0.8)	2.4(2.8)	0.8(0.8)
missing	0	0	0	2
**Kirkpatrick tool Level 1 (Reaction) and Level 2 (Learning) evaluation**				
I am confident with identifying the Long Saphenous Vein (LSV) using ultrasound				
Median (IQR)	5.0 [5.0, 5.0]	5.0 [5.0, 5.0]	5.0 [5.0, 5.0]	5.0 [5.0, 5.0]
Mean (SD)	4.9(0.2)	5.0(0.2)	4.9(0.3)	5.0(0.0)
missing	0	0	0	2
I understand the Anatomy & Physiology of the LSV				
Median (IQR)	5.0 [5.0, 5.0]	5.0 [5.0, 5.0]	5.0 [5.0, 5.0]	5.0 [5.0, 5.0]
Mean (SD)	4.9(0.4)	5.0(0.2)	4.9(0.2)	4.8(0.4)
missing	0	0	0	2
I am confident to make an incision and set up for harvest LSV				
Median (IQR)	5.0 [4.8, 5.0]	5.0 [5.0, 5.0]	5.0 [4.0, 5.0]	5.0 [5.0, 5.0]
Mean (SD)	4.7(0.6)	5.0(0.0)	4.6(0.7)	5.0(0.0)
missing	0	0	0	2
I am confident to dissect the LSV with minimal help				
Median (IQR)	4.0 [3.0, 5.0]	5.0 [5.0, 5.0]	4.0 [3.0, 5.0]	5.0 [5.0, 5.0]
Mean (SD)	4.1(0.9)	4.8(0.4)	3.9(1.3)	5.0(0.0)
missing	0	0	0	2
I am confident to ligate and coagulate the LSV with minimal help				
Median (IQR)	4.0 [3.0, 5.0]	5.0 [4.0, 5.0]	4.0 [2.5, 5.0]	5.0 [5.0, 5.0]
Mean (SD)	4.0(0.9)	4.6(0.5)	3.5(1.3)	5.0(0.0)
missing	0	0	0	2
I am confident about the management of complications during harvesting				
Median (IQR)	4.0 [3.0, 4.0]	4.0 [4.0, 5.0]	3.0 [2.0, 5.0]	5.0 [5.0, 5.0]
Mean (SD)	3.6(0.6)	4.3(0.5)	3.5(1.4)	5.0(0.0)
missing	0	0	0	2
I am confident to ask for help when required				
Median (IQR)	5.0 [5.0, 5.0]	5.0 [5.0, 5.0]	5.0 [5.0, 5.0]	5.0 [5.0, 5.0]
Mean (SD)	4.7(0.6)	5.0(0.0)	4.9(0.3)	5.0(0.0)
missing	0	0	0	2

### Kirkpatrick evaluation

Confidence scores across Kirkpatrick domains were high for anatomical knowledge and procedural step-up median [IQR] 5.0 [5.0, 5.0] during and post training. For advanced tasks (dissection, ligation, coagulation and management of complications) confidence increased from median 3.0 to 5.0 during training to 4.0–5.0 post-training across all trainees. Confidence in recognising when to see assistance was uniformly high ( Table 3).

### Patient outcomes

Of 46 patients, 40 (87%) were male. Median age was 62.0–68.5 years across all groups.

### Respiratory parameters

Overall, across four trainees (46 patients) median PCO _2_ increased from baseline to 60 minutes, accompanied by increases in ETCO _2_ at 60 minutes in all three trainees. Median pH remained stable in T1 and decreased from 7.4 at baseline to 7.3 at 60 minutes in the rest of the trainees. Minute volume increased over time in T1 and T4, remained stable in T2 and decreased at 60 minutes in T4 ( Table 4).

**Table 4.** illustrates Carbon dioxide (CO2) and respiratory parameters at baseline, during harvest and post-harvest following Endoscopic Vein Harvesting training.

	Trainee 1 (T1) (ST group)			T2 (ST group)			T4 (CT group)		
Characteristic	Baseline n = 20	Half Hour n = 20	One Hour n = 20	Baseline n = 20	Half Hour n = 20	One Hour n = 20	Baseline n = 5	Half Hour n = 5	One Hour n = 5
**Age**									
Median [IQR]	62.0 [54.5, 68.0]	62.0 [54.5, 68.0]	62.0 [54.5, 68.0]	68.5 [59.0, 76.0]	68.5 [59.0, 76.0]	68.5 [59.0, 76.0]	68.0 [56.0, 76.0]	68.0 [56.0, 76.0]	68.0 [56.0, 76.0]
**pH levels**									
Median [IQR]	7.4 [7.4, 7.4]	7.4 [7.3, 7.4]	7.4 [7.4, 7.4]	7.4 [7.4, 7.5]	7.4 [7.3, 7.4]	7.3 [7.3, 7.4]	7.4 [7.3, 7.4]	0.0 [0.0, 0.0]	7.3 [7.3, 7.3]
**Partial Pressure of Carbon di-oxide (pCO2)**									
Median [IQR]	5.6 [5.0, 6.0]	5.7 [5.3, 6.3]	5.8 [5.3, 6.0]	5.2 [5.0, 5.5]	6.0 [5.4, 6.9]	6.3 [5.6, 7.0]	5.6 [4.8, 5.9]	0.0 [0.0, 0.0]	6.5 [5.3, 6.5]
**Partial Pressure of Oxygen (PO2)**									
Median [IQR]	23.8 [13.9, 33.3]	26.8 [18.7, 30.0]	25.6 [19.5, 31.3]	24.7 [20.4, 36.1]	26.0 [20.0, 33.5]	26.7 [20.6, 31.3]	29.4 [25.3, 33.9]	0.0 [0.0, 0.0]	28.1 [28.1, 37.5]
**End-Tidal Carbon Dioxide (ETCO2)**									
Median [IQR]	4.5 [4.0, 4.7]	4.6 [4.1, 4.9]	4.6 [4.0, 5.2]	4.3 [3.9, 4.8]	4.6 [4.3, 5.6]	4.8 [4.1, 5.6]	4.7 [4.6, 4.9]	4.6 [4.5, 4.8]	6.2 [5.6, 6.3]
**Minute volume**									
Median [IQR]	6.5 [6.0, 7.6]	7.0 [6.2, 7.7]	7.2 [6.1, 7.6]	6.7 [6.1, 7.0]	6.7 [6.2, 7.4]	6.7 [6.4, 8.2]	5.1 [5.1, 5.2]	5.2 [5.1, 5.2]	4.5 [4.3, 4.9]

### Wound outcomes

Wound complications were assessed at Days 1, 3, and 5 and sensory symptoms at six weeks. Bruising was more frequent in T2 than T1 at all early time points (Day 1: 45.0% vs 15.0%; Day 3: 45.0% vs 30.0%; Day 5: 45.0% vs 35.0%). All patients in T3 (n = 1) and T4 (n = 5) exhibited bruising. Hematoma was uncommon overall, observed only in isolated cases in T1 and T2 (5.0% at any time point). At six weeks, leg numbness was reported in 40.0% of T1 and 25.0% of T2 patients and in all patients in T3 and T4. Leg tenderness was more common in T1 (65.0%) compared with T2 (25.0%) and was reported in all T3 patients and 60.0% of T4 patients ( Table 3).

### Major adverse cardiac events

MACE was assessed up to 12 months and survival to 24 months. Repeat angina was infrequent occurring in 5.0% of T1 patients at 3 and 6 months and in 5.0% of T2 patients at 12 months. No cases were reported in T3 or T4. Re-intervention was reported in T1 at 1 and 9 months (each 5.0%) and in T2 at 1 month (5.0%) with none in T3 or T4. Myocardial infarction occurred only in T1 (5.0% at 9 months). Survival in T1 was 100.0% to 9 months and 95.0% at 12 and 24 months. T2 and T4 demonstrated 100.0% survival throughout follow-up while T3 survival was 100.0% at 1 month.

### CT angiography

CT angiography was scheduled at 12–24 months in which ST group (21/39 patients) and CT group (3/6) underwent imaging. One patient died at 11 months due to subdural haematoma and 18 did not attend due to work commitments. In the CT group, one did not attend following stroke and two were unable to undergo imaging due to lack of radiology support. All scans were independently reviewed by a cardiologist and a surgeon. One symptomatic patient underwent coronary angiogram at 6 months, all grafts patent.

Among asymptomatic patients, 13 vein grafts (T1), 23 (T2) and 2 (T4) were assessed. Flow-limiting lesions identified in 1/13 grafts (T1) and 4/23 (T2). Occlusion occurred in 2/13 (T1) and 8/23 (T2) and the remaining grafts were patent. Affected territories included Diagonal (D1) (2/13 T1; 5/23 T2), Obtuse Marginal (OM2) (1/13 T1; 3/23 T2), and Right Coronary Artery (RCA/PDA) (0/13 T1; 4/23 T2). All lesions in T1 and T2 were classified as ACC/AHA Type C and small-calibre diabetic vessels were reported in 7 (T1) and 14 (T2) ( Table 5).

**Table 5.** illustrates the incidence of postoperative complications, Major Adverse Cardiac Events (MACE) and other clinical investigations.

Variable	T1 (n-20)	T2 (n = 20)	T3 (n = 1)	T4 (n = 5)
Leg wound numbness, n(%) 6 weeks	8 (40.0)	5 (25.0)	1 (100.0)	5 (100.0)
Leg wound tenderness, n(%) 6 weeks	13 (65.0)	5 (25.0)	1(100.0)	3 (60.0)
Arrythmias, n(%)	4 (21.0)	1 (5.0)	NA	NA
missing	0	0	1	5
Atrial fibrillation, n(%)	5 (25.0)	1 (5.0)	NA	1 (20.0)
missing	0	0	0	0
Post- surgery coronary angiogram, n(%)	1 (5.0)	0 (0.0)	0 (0.0)	0 (0.0)
Investigations done for non-cardiac surgery, n(%)	5 (26.0)	4 (20.0)	0 (0.0)	0 (0.0)
**Repeat angina, n(%)**				
1 month	0 (0.0)	0 (0.0)	0 (0.0)	0 (0.0)
3 months	1 (5.0)	0 (0.0)	0 (0.0)	0 (0.0)
6 months	1 (5.0)	0 (0.0)	0 (0.0)	0 (0.0)
9 months	0 (0.0)	0 (0.0)	0 (0.0)	0 (0.0)
12 months	0 (0.0)	1 (5.0)	0 (0.0)	0 (0.0)
Missing data	1	1	0	0
**Breathlessness, n(%)**				
1 month	5 (25.0)	5 (25.0)	0 (0.0)	4 (80.0)
3 months	3 (15.0)	4 (20.0)	NA	1 (20.0)
6 months	3 (15.0)	3 (15.0)	NA	0 (0.0)
9 months	0 (0.0)	3 (15.0)	NA	0 (0.0)
12 months	1 (5.0)	2 (11.0)	NA	0 (0.0)
Missing data	1	3	1	0
**Re-intervention, n(%)**				
1 month	1 (5.0)	1 (5.0)	0 (0.0)	0 (0.0)
3 months	0 (0.0)	0 (0.0)	NA	0 (0.0)
6 months	0 (0.0)	0 (0.0)	NA	0 (0.0)
9 months	1 (5.0)	0 (0.0)	NA	0 (0.0)
12 months	0 (0.0)	0 (0.0)	NA	0 (0.0)
Missing data	1	0	1	0
**Myocardial infarction, n(%)**				
1 month	0 (0.0)	0 (0.0)	0 (0.0)	0 (0.0)
3 months	0 (0.0)	0 (0.0)	NA	0 (0.0)
6 months	0 (0.0)	0 (0.0)	NA	0 (0.0)
9 months	1 (5.0)	0 (0.0)	NA	0 (0.0)
12 months	0 (0.0)	0 (0.0)	NA	0 (0.0)
Missing data	1	0	1	0
**Survival, n(%)**				
1 month	20 (100.0)	20 (100.0)	1 (100.0)	5 (100.0)
3 months	20 (100.0)	20 (100.0)	NA	5 (100.0)
6 months	20 (100.0)	20 (100.0)	NA	5 (100.0)
9 months	20 (100.0)	20 (100.0)	NA	5 (100.0)
12 months	19 (95.0)	20 (100.0)	NA	5 (100.0)
24 months	19 (95.0)	20 (100.0)	NA	5 (100.0)
Missing data	0	0	1	0
**Cause of Major Adverse Cardiac Events (MACEs) at 24 months**				
CT contrast angiography (n = 24/45)	7/20	14/20	NA	3/5
Total number of vein grafts assessed (n = 38)	13	23	NA	2
Vein graft patent	10/13	11/23		2/2
Flow-limiting (n = 5/36)	1/13	4/23	NA	0
Completely blocked (n = 10/36)	2/13	8/23	NA	0
Diagonal graft (n = 7/36)	2/13	5/23	NA	0
Obtuse Marginal graft (OM2)(n = 4/36)	1/13	3/23	NA	0
Right Coronary Artery (Posterior Descending Artery) (n = 4/36)	0/13	4/23	NA	0
ACC/AHA scoring for coronary artery stenosis: Type C (diffuse >2 cm) lesion	7 patients	14 patients	NA	0
Small-calibre diabetic vessels	7 patients	14 patients	NA	0

ACC/AHA (American College of Cardiology/American Heart Association); CT (Computed Tomography); NA (not available).

### Patient reported outcomes

Wound satisfaction was high (median 98–100). EQ-5D-5L domains improved post-operatively, with most patients reporting “no problem” across mobility, self-care, usual activities, pain, and anxiety ( Table 6).

**Table 6.** illustrates Short Form-36 (SF-36) outcomes measured preoperatively, postoperatively and at 1-year follow-up.

	Structured Training (T1 & T2)				Conventional Training (T4)	
Characteristics	T1		T2		T4	
	Pre-surgery (n = 20)	Post-surgery (n = 20)	Pre-surgery (n = 20)	Post-surgery (n = 20)	Pre-surgery (n = 5)	Post-surgery (n = 5)
SF-36 questionnaire						
**Compared to one year ago how would you rate your health in general now, n (%)**						
Much better	1.0 (5.0%)	10.0 (55.6%)	0.0 (0.0%)	11.0 (55.0%)	0.0 (0.0%)	2.0 (40.0%)
Somewhat better	2.0 (10.0%)	8.0 (44.4%)	2.0 (10.0%)	6.0 (30.0%)	0.0 (0.0%)	1.0 (20.0%)
About the same	8.0 (40.0%)	0.0 (0.0%)	6.0 (30.0%)	2.0 (10.0%)	1.0 (20.0%)	1.0 (20.0%)
Somewhat worse	7.0 (35.0%)	0.0 (0.0%)	9.0 (45.0%)	1.0 (5.0%)	1.0 (20.0%)	1.0 (20.0%)
Much worse	2.0 (10.0%)	0.0 (0.0%)	3.0 (15.0%)	0.0 (0.0%)	3.0 (60.0%)	0.0 (0.0%)
missing	0	2	0	0	0	0
**Vigorous Activities such as running lifting heavy objects participating in strenuous sports, n (%)**						
No, not limited at all	1.0 (5.0%)	7.0 (38.9%)	4.0 (20.0%)	9.0 (45.0%)	0.0 (0.0%)	3.0 (60.0%)
Yes, limited a little	7.0 (35.0%)	10.0 (55.6%)	1.0 (5.0%)	9.0 (45.0%)	2.0 (40.0%)	1.0 (20.0%)
Yes, limited a lot	12.0 (60.0%)	1.0 (5.6%)	15.0 (75.0%)	2.0 (10.0%)	3.0 (60.0%)	1.0 (20.0%)
missing	0	2	0	0	0	0
**Moderate Activities such as moving a table pushing a vacuum cleaner bowling or playing golf or any other sports, n (%)**						
No, not limited at all	2.0 (10.0%)	14.0 (77.8%)	5.0 (25.0%)	16.0 (80.0%)	0.0 (0.0%)	4.0 (80.0%)
Yes, limited a little	13.0 (65.0%)	4.0 (22.2%)	6.0 (30.0%)	4.0 (20.0%)	2.0 (50.0%)	1.0 (20.0%)
Yes, limited a lot	5.0 (25.0%)	0.0 (0.0%)	9.0 (45.0%)	0.0 (0.0%)	2.0 (50.0%)	0.0 (0.0%)
missing	0	2	0	0	1	0
**Lifting or carrying groceries**						
No, not limited at all	5.0 (26.3%)	16.0 (88.9%)	8.0 (40.0%)	19.0 (95.0%)	0.0 (0.0%)	3.0 (60.0%)
Yes, limited a little	10.0 (52.6%)	2.0 (11.1%)	4.0 (20.0%)	1.0 (5.0%)	1.0 (20.0%)	2.0 (40.0%)
Yes, limited a lot	4.0 (21.1%)	0.0 (0.0%)	8.0 (40.0%)	0.0 (0.0%)	4.0 (80.0%)	0.0 (0.0%)
missing	1	2	0	0	0	0
**Climbing several flights of stairs**						
No, not limited at all	2.0 (10.0%)	14.0 (77.8%)	6.0 (30.0%)	13.0 (65.0%)	0.0 (0.0%)	3.0 (60.0%)
Yes, limited a little	12.0 (60.0%)	4.0 (22.2%)	4.0 (20.0%)	6.0 (30.0%)	3.0 (60.0%)	2.0 (40.0%)
Yes, limited a lot	6.0 (30.0%)	0.0 (0.0%)	10.0 (50.0%)	1.0 (5.0%)	2.0 (40.0%)	0.0 (0.0%)
missing	0	2	0	0	0	0
**Climbing one flights of stairs**						
No, not limited at all	8.0 (40.0%)	17.0 (94.4%)	7.0 (35.0%)	16.0 (80.0%)	0.0 (0.0%)	2.0 (40.0%)
Yes, limited a little	7.0 (35.0%)	1.0 (5.6%)	6.0 (30.0%)	3.0 (15.0%)	3.0 (75.0%)	3.0 (60.0%)
Yes, limited a lot	5.0 (25.0%)	0.0 (0.0%)	7.0 (35.0%)	1.0 (5.0%)	1.0 (25.0%)	0.0 (0.0%)
missing	0	2	0	0	1	0
**Bending Kneeling or stooping**						
No, not limited at all	6.0 (30.0%)	17.0 (94.4%)	10.0 (50.0%)	17.0 (85.0%)	1.0 (25.0%)	3.0 (60.0%)
Yes, limited a little	10.0 (50.0%)	1.0 (5.6%)	4.0 (20.0%)	3.0 (15.0%)	1.0 (25.0%)	2.0 (40.0%)
Yes, limited a lot	4.0 (20.0%)	0.0 (0.0%)	6.0 (30.0%)	0.0 (0.0%)	2.0 (50.0%)	0.0 (0.0%)
missing	0	2	0	0	1	0
**Walking more than a mile**						
No, not limited at all	3.0 (15.0%)	14.0 (77.8%)	3.0 (15.0%)	13.0 (65.0%)	0.0 (0.0%)	2.0 (40.0%)
Yes, limited a little	11.0 (55.0%)	4.0 (22.2%)	3.0 (15.0%)	6.0 (30.0%)	3.0 (60.0%)	3.0 (60.0%)
Yes, limited a lot	6.0 (30.0%)	0.0 (0.0%)	14.0 (70.0%)	1.0 (5.0%)	2.0 (40.0%)	0.0 (0.0%)
missing	0	2	0	0	0	0
**Walking several blocks**						
No, not limited at all	2.0 (10.0%)	17.0 (94.4%)	5.0 (25.0%)	16.0 (80.0%)	0.0 (0.0%)	2.0 (40.0%)
Yes, limited a little	12.0 (60.0%)	1.0 (5.6%)	5.0 (25.0%)	3.0 (15.0%)	3.0 (60.0%)	3.0 (60.0%)
Yes, limited a lot	6.0 (30.0%)	0.0 (0.0%)	10.0 (50.0%)	1.0 (5.0%)	2.0 (40.0%)	0.0 (0.0%)
missing	0	2	0	0	0	0
**Walking one block**						
No, not limited at all	8.0 (40.0%)	17.0 (94.4%)	8.0 (40.0%)	18.0 (90.0%)	0.0 (0.0%)	3.0 (60.0%)
Yes, limited a little	7.0 (35.0%)	1.0 (5.6%)	5.0 (25.0%)	1.0 (5.0%)	2.0 (40.0%)	2.0 (40.0%)
Yes, limited a lot	5.0 (25.0%)	0.0 (0.0%)	7.0 (35.0%)	1.0 (5.0%)	3.0 (60.0%)	0.0 (0.0%)
missing	0	2	0	0	0	0
**Bathing or dressing myself**						
No, not limited at all	13.0 (68.4%)	17.0 (94.4%)	14.0 (70.0%)	18.0 (90.0%)	1.0 (25.0%)	4.0 (80.0%)
Yes, limited a little	4.0 (21.1%)	1.0 (5.6%)	2.0 (10.0%)	2.0 (10.0%)	1.0 (25.0%)	1.0 (20.0%)
Yes, limited a lot	2.0 (10.5%)	0.0 (0.0%)	4.0 (20.0%)	0.0 (0.0%)	2.0 (50.0%)	0.0 (0.0%)
Missing	1	2	0	0	1	0
**During the past 4 weeks have you had any of the following problems with your work or other regular daily activities as a result of your physical health, n (%)**						
Cut down the amount of time you spent on work or other activities	18.0 (90.0%)	0.0 (0.0%)	17.0 (85.0%)	5.0 (25.0%)	5.0 (100.0%)	2.0 (40.0%)
missing	0	2	0	0	0	0
Accomplished less than you would like	18.0 (90.0%)	0.0 (0.0%)	17.0 (85.0%)	6.0 (30.0%)	5.0 (100.0%)	2.0 (40.0%)
missing	0	2	0	0	0	0
Were limited in the kind of work or other activities	19.0 (95.0%)	0.0 (0.0%)	16.0 (80.0%)	3.0 (15.0%)	5.0 (100.0%)	2.0 (40.0%)
missing	0	2	0	0	0	0
Had difficulty performing the work or other activities	17.0 (85.0%)	1.0 (5.6%)	16.0 (80.0%)	4.0 (20.0%)	5.0 (100.0%)	2.0 (40.0%)
missing	0	2	0	0	0	0
**During the past 4 weeks have you had any of the following problems with your work or other regular daily activities as a result of any emotional problems such as feeling depressed or anxious, n (%)**						
Cut down the amount of time you spent on work or other activities	18.0 (90.0%)	0.0 (0.0%)	15.0 (75.0%)	3.0 (15.0%)	2.0 (40.0%)	0.0 (0.0%)
missing	0	2	0	0	0	0
Accomplished less than you like	17.0 (85.0%)	1.0 (5.6%)	15.0 (75.0%)	4.0 (20.0%)	3.0 (75.0%)	0.0 (0.0%)
missing	0	2			1	0
Didn’t do work or other activities as carefully as usual	17.0 (85.0%)	1.0 (5.6%)	14.0 (70.0%)	2.0 (10.0%)	3.0 (75.0%)	0.0 (0.0%)
Missing	0	2	0	0	1	0
**During the past 4 weeks to what extent has your physical health or emotional problems interfered with your normal social activities with family friends neighbours or groups, n (%)**						
Not at all	2.0 (10.0%)	14.0 (87.5%)	5.0 (25.0%)	13.0 (65.0%)	0.0 (0.0%)	4.0 (80.0%)
slightly	7.0 (35.0%)	2.0 (12.5%)	1.0 (5.0%)	6.0 (30.0%)	2.0 (40.0%)	1.0 (20.0%)
moderately	4.0 (20.0%)	0.0 (0.0%)	5.0 (25.0%)	0.0 (0.0%)	1.0 (20.0%)	0.0 (0.0%)
quite a bit	3.0 (15.0%)	0.0 (0.0%)	4.0 (20.0%)	1.0 (5.0%)	0.0 (0.0%)	0.0 (0.0%)
extremely	4.0 (20.0%)	0.0 (0.0%)	5.0 (25.0%)	0.0 (0.0%)	2.0 (40.0%)	0.0 (0.0%)
missing	0	4	0	0	0	0
**How much bodily pain have you had during the past 4 weeks**						
None	6.0 (30.0%)	13.0 (72.2%)	4.0 (20.0%)	12.0 (60.0%)	0.0 (0.0%)	0.0 (0.0%)
very mild	2.0 (10.0%)	5.0 (27.8%)	1.0 (5.0%)	5.0 (25.0%)	0.0 (0.0%)	3.0 (60.0%)
mild	3.0 (15.0%)	0.0 (0.0%)	3.0 (15.0%)	1.0 (5.0%)	1.0 (20.0%)	1.0 (20.0%)
moderate	5.0 (25.0%)	0.0 (0.0%)	4.0 (20.0%)	1.0 (5.0%)	2.0 (40.0%)	0.0 (0.0%)
severe	4.0 (20.0%)	0.0 (0.0%)	8.0 (40.0%)	1.0 (5.0%)	2.0 (40.0%)	1.0 (20.0%)
very severe	0.0 (0.0%)	0.0 (0.0%)	0.0 (0.0%)	0.0 (0.0%)	0.0 (0.0%)	0.0 (0.0%)
missing	0	2	0	0	0	0
**During the past 4 weeks how much did pain interfere with your normal work including both work outside the home and housework, n (%)**						
Not at all	7.0 (35.0%)	17.0 (94.4%)	5.0 (25.0%)	14.0 (70.0%)	0.0 (0.0%)	1.0 (20.0%)
slightly	1.0 (5.0%)	1.0 (5.6%)	3.0 (15.0%)	5.0 (25.0%)	0.0 (0.0%)	3.0 (60.0%)
moderately	5.0 (25.0%)	0.0 (0.0%)	5.0 (25.0%)	0.0 (0.0%)	1.0 (20.0%)	0.0 (0.0%)
quite a bit	5.0 (25.0%)	0.0 (0.0%)	5.0 (25.0%)	1.0 (5.0%)	4.0 (80.0%)	1.0 (20.0%)
extremely	2.0 (10.0%)	0.0 (0.0%)	2.0 (10.0%)	0.0 (0.0%)	0.0 (0.0%)	0.0 (0.0%)
missing	0	2	0	0	0	0
**How much during the past 4 weeks Did you feel full of pep**						
None of the time	2.0 (10.0%)	13.0 (72.2%)	4.0 (21.1%)	14.0 (73.7%)	3.0 (60.0%)	4.0 (80.0%)
a little of the time	1.0 (5.0%)	3.0 (16.7%)	3.0 (15.8%)	2.0 (10.5%)	0.0 (0.0%)	1.0 (20.0%)
some of the time	8.0 (40.0%)	2.0 (11.1%)	3.0 (15.8%)	2.0 (10.5%)	1.0 (20.0%)	0.0 (0.0%)
a good bit of the time	5.0 (25.0%)	0.0 (0.0%)	3.0 (15.8%)	0.0 (0.0%)	1.0 (20.0%)	0.0 (0.0%)
most of the time	4.0 (20.0%)	0.0 (0.0%)	3.0 (15.8%)	1.0 (5.3%)	0.0 (0.0%)	0.0 (0.0%)
all the time	0.0 (0.0%)	0.0 (0.0%)	3.0 (15.8%)	0.0 (0.0%)	0.0 (0.0%)	0.0 (0.0%)
missing	0	2	1	1	0	0
**Have you been a very nervous person**						
None of the time	6.0 (30.0%)	11.0 (61.1%)	5.0 (26.3%)	11.0 (57.9%)	2.0 (40.0%)	4.0 (80.0%)
a little of the time	5.0 (25.0%)	5.0 (27.8%)	4.0 (21.1%)	6.0 (31.6%)	2.0 (40.0%)	1.0 (20.0%)
some of the time	4.0 (20.0%)	1.0 (5.6%)	3.0 (15.8%)	0.0 (0.0%)	1.0 (20.0%)	0.0 (0.0%)
a good bit of the time	2.0 (10.0%)	1.0 (5.6%)	2.0 (10.5%)	0.0 (0.0%)	0.0 (0.0%)	0.0 (0.0%)
most of the time	3.0 (15.0%)	0.0 (0.0%)	2.0 (10.5%)	2.0 (10.5%)	0.0 (0.0%)	0.0 (0.0%)
all the time	0.0 (0.0%)	0.0 (0.0%)	3.0 (15.8%)	0.0 (0.0%)	0.0 (0.0%)	0.0 (0.0%)
missing	0	2	1	1	0	0
**Have you felt so down in the dumps that nothing could cheer you up**						
None of the time	11.0 (55.0%)	15.0 (83.3%)	5.0 (26.3%)	13.0 (68.4%)	5.0 (100.0%)	3.0 (60.0%)
a little of the time	2.0 (10.0%)	2.0 (11.1%)	5.0 (26.3%)	3.0 (15.8%)	0.0 (0.0%)	2.0 (40.0%)
some of the time	2.0 (10.0%)	1.0 (5.6%)	3.0 (15.8%)	1.0 (5.3%)	0.0 (0.0%)	0.0 (0.0%)
a good bit of the time	1.0 (5.0%)	0.0 (0.0%)	2.0 (10.5%)	1.0 (5.3%)	0.0 (0.0%)	0.0 (0.0%)
most of the time	4.0 (20.0%)	0.0 (0.0%)	1.0 (5.3%)	1.0 (5.3%)	0.0 (0.0%)	0.0 (0.0%)
all the time	0.0 (0.0%)	0.0 (0.0%)	3.0 (15.8%)	0.0 (0.0%)	0.0 (0.0%)	0.0 (0.0%)
missing	0	2	1	1	0	0
**Have you felt calm and peaceful**						
None of the time	2.0 (10.0%)	0.0 (0.0%)	1.0 (5.3%)	1.0 (5.3%)	0.0 (0.0%)	0.0 (0.0%)
a little of the time	4.0 (20.0%)	0.0 (0.0%)	4.0 (21.1%)	1.0 (5.3%)	1.0 (20.0%)	0.0 (0.0%)
some of the time	4.0 (20.0%)	1.0 (5.6%)	1.0 (5.3%)	1.0 (5.3%)	1.0 (20.0%)	1.0 (20.0%)
a good bit of the time	6.0 (30.0%)	2.0 (11.1%)	3.0 (15.8%)	1.0 (5.3%)	0.0 (0.0%)	0.0 (0.0%)
most of the time	3.0 (15.0%)	14.0 (77.8%)	5.0 (26.3%)	13.0 (68.4%)	3.0 (60.0%)	4.0 (80.0%)
all the time	1.0 (5.0%)	1.0 (5.6%)	5.0 (26.3%)	2.0 (10.5%)	0.0 (0.0%)	0.0 (0.0%)
missing	0	2	1	1	0	0
**Did you have a lot of energy**						
None of the time	2.0 (10.0%)	0.0 (0.0%)	0.0 (0.0%)	0.0 (0.0%)	2.0 (40.0%)	0.0 (0.0%)
a little of the time	4.0 (20.0%)	2.0 (11.1%)	8.0 (42.1%)	2.0 (10.5%)	1.0 (20.0%)	1.0 (20.0%)
some of the time	4.0 (20.0%)	2.0 (11.1%)	2.0 (10.5%)	1.0 (5.3%)	2.0 (40.0%)	1.0 (20.0%)
a good bit of the time	5.0 (25.0%)	6.0 (33.3%)	2.0 (10.5%)	4.0 (21.1%)	0.0 (0.0%)	0.0 (0.0%)
most of the time	4.0 (20.0%)	8.0 (44.4%)	3.0 (15.8%)	10.0 (52.6%)	0.0 (0.0%)	2.0 (40.0%)
all the time	1.0 (5.0%)	0.0 (0.0%)	4.0 (21.1%)	2.0 (10.5%)	0.0 (0.0%)	1.0 (20.0%)
missing	0	2	1	1	0	0
**Have you felt downhearted and blue**						
None of the time	9.0 (45.0%)	13.0 (72.2%)	5.0 (26.3%)	12.0 (63.2%)	1.0 (20.0%)	4.0 (80.0%)
A little of the time	5.0 (25.0%)	5.0 (27.8%)	6.0 (31.6%)	5.0 (26.3%)	4.0 (80.0%)	0.0 (0.0%)
Some of the time	3.0 (15.0%)	0.0 (0.0%)	3.0 (15.8%)	1.0 (5.3%)	0.0 (0.0%)	1.0 (20.0%)
A good bit of the time	0.0 (0.0%)	0.0 (0.0%)	1.0 (5.3%)	1.0 (5.3%)	0.0 (0.0%)	0.0 (0.0%)
Most of the time	3.0 (15.0%)	0.0 (0.0%)	1.0 (5.3%)	0.0 (0.0%)	0.0 (0.0%)	0.0 (0.0%)
All the time	0.0 (0.0%)	0.0 (0.0%)	3.0 (15.8%)	0.0 (0.0%)	0.0 (0.0%)	0.0 (0.0%)
missing	0	2	1	1	0	0
**Did you feel worn out**						
None of the time	2.0 (10.0%)	3.0 (16.7%)	1.0 (5.3%)	10.0 (52.6%)	1.0 (20.0%)	3.0 (60.0%)
A little of the time	2.0 (10.0%)	15.0 (83.3%)	3.0 (15.8%)	6.0 (31.6%)	1.0 (20.0%)	1.0 (20.0%)
Some of the time	8.0 (40.0%)	0.0 (0.0%)	7.0 (36.8%)	2.0 (10.5%)	0.0 (0.0%)	0.0 (0.0%)
A good bit of the time	2.0 (10.0%)	0.0 (0.0%)	2.0 (10.5%)	1.0 (5.3%)	1.0 (20.0%)	0.0 (0.0%)
Most of the time	5.0 (25.0%)	0.0 (0.0%)	3.0 (15.8%)	0.0 (0.0%)	1.0 (20.0%)	1.0 (20.0%)
All the time	1.0 (5.0%)	0.0 (0.0%)	3.0 (15.8%)	0.0 (0.0%)	1.0 (20.0%)	0.0 (0.0%)
missing	0	2	1	1	0	0
**Have you been a happy person**						
None of the time	0.0 (0.0%)	1.0 (5.6%)	0.0 (0.0%)	0.0 (0.0%)	0.0 (0.0%)	0.0 (0.0%)
A little of the time	0.0 (0.0%)	0.0 (0.0%)	1.0 (5.3%)	0.0 (0.0%)	0.0 (0.0%)	0.0 (0.0%)
Some of the time	2.0 (10.0%)	0.0 (0.0%)	2.0 (10.5%)	1.0 (5.6%)	0.0 (0.0%)	0.0 (0.0%)
A good bit of the time	3.0 (15.0%)	2.0 (11.1%)	3.0 (15.8%)	3.0 (16.7%)	1.0 (20.0%)	1.0 (20.0%)
Most of the time	9.0 (45.0%)	13.0 (72.2%)	4.0 (21.1%)	12.0 (66.7%)	4.0 (80.0%)	3.0 (60.0%)
All the time	6.0 (30.0%)	2.0 (11.1%)	9.0 (47.4%)	2.0 (11.1%)	0.0 (0.0%)	1.0 (20.0%)
missing	0	2	1	2	0	0
**Did you feel tired**						
None of the time	1.0 (5.0%)	0.0 (0.0%)	1.0 (5.3%)	4.0 (21.1%)	0.0 (0.0%)	2.0 (40.0%)
A little of the time	2.0 (10.0%)	16.0 (88.9%)	2.0 (10.5%)	10.0 (52.6%)	2.0 (40.0%)	3.0 (60.0%)
Aome of the time	5.0 (25.0%)	1.0 (5.6%)	4.0 (21.1%)	2.0 (10.5%)	1.0 (20.0%)	0.0 (0.0%)
A good bit of the time	5.0 (25.0%)	0.0 (0.0%)	3.0 (15.8%)	1.0 (5.3%)	0.0 (0.0%)	0.0 (0.0%)
Most of the time	5.0 (25.0%)	1.0 (5.6%)	5.0 (26.3%)	1.0 (5.3%)	2.0 (40.0%)	0.0 (0.0%)
All the time	2.0 (10.0%)	0.0 (0.0%)	4.0 (21.1%)	1.0 (5.3%)	0.0 (0.0%)	0.0 (0.0%)
missing	0	2	1	1	0	0
**During the past 4 weeks: how much of the time has your physical health or emotional problems interfered with your social activities like visiting with friends, relatives etc, n (%)**						
All of the time	1.0 (5.0%)	0.0 (0.0%)	3.0 (15.8%)	0.0 (0.0%)	2.0 (40.0%)	0.0 (0.0%)
Most of the time	5.0 (25.0%)	0.0 (0.0%)	6.0 (31.6%)	2.0 (11.1%)	2.0 (40.0%)	0.0 (0.0%)
Some of the time	4.0 (20.0%)	1.0 (5.9%)	3.0 (15.8%)	1.0 (5.6%)	1.0 (20.0%)	1.0 (20.0%)
A little of the time	5.0 (25.0%)	3.0 (17.6%)	4.0 (21.1%)	3.0 (16.7%)	0.0 (0.0%)	1.0 (20.0%)
None of the time	5.0 (25.0%)	13.0 (76.5%)	3.0 (15.8%)	12.0 (66.7%)	0.0 (0.0%)	3.0 (60.0%)
missing	0	3	1	2	0	0
**How TRUE or FALSE is each of the following statement for you, n (%)**						
**I seem to get sick a little easier than other people**						
Definitely true	1.0 (5.0%)	0.0 (0.0%)	1.0 (5.0%)	0.0 (0.0%)	0.0 (0.0%)	0.0 (0.0%)
Mostly true	2.0 (10.0%)	1.0 (5.6%)	3.0 (15.0%)	3.0 (15.0%)	1.0 (20.0%)	0.0 (0.0%)
Don’t know	1.0 (5.0%)	3.0 (16.7%)	4.0 (20.0%)	5.0 (25.0%)	2.0 (40.0%)	2.0 (40.0%)
Mostly false	5.0 (25.0%)	5.0 (27.8%)	5.0 (25.0%)	11.0 (55.0%)	2.0 (40.0%)	1.0 (20.0%)
Definitely false	11.0 (55.0%)	9.0 (50.0%)	7.0 (35.0%)	1.0 (5.0%)	0.0 (0.0%)	2.0 (40.0%)
missing	0	2	0	0	0	0
**I am as healthy as anybody I know**						
Definitely true	10.0 (50.0%)	10.0 (55.6%)	2.0 (10.0%)	1.0 (5.0%)	0.0 (0.0%)	0.0 (0.0%)
Mostly true	3.0 (15.0%)	5.0 (27.8%)	6.0 (30.0%)	14.0 (70.0%)	0.0 (0.0%)	1.0 (20.0%)
Don’t know	5.0 (25.0%)	2.0 (11.1%)	7.0 (35.0%)	1.0 (5.0%)	3.0 (60.0%)	2.0 (40.0%)
Mostly false	2.0 (10.0%)	1.0 (5.6%)	3.0 (15.0%)	4.0 (20.0%)	1.0 (20.0%)	2.0 (40.0%)
Definitely false	0.0 (0.0%)	0.0 (0.0%)	2.0 (10.0%)	0.0 (0.0%)	1.0 (20.0%)	0.0 (0.0%)
Missing	0	2	0	0	0	0
**I expect my health to get worse**						
Definitely true	3.0 (15.0%)	1.0 (5.6%)	1.0 (5.0%)	0.0 (0.0%)	1.0 (20.0%)	1.0 (20.0%)
Mostly true	1.0 (5.0%)	1.0 (5.6%)	5.0 (25.0%)	15.0 (75.0%)	0.0 (0.0%)	1.0 (20.0%)
Don’t know	2.0 (10.0%)	5.0 (27.8%)	4.0 (20.0%)	2.0 (10.0%)	1.0 (20.0%)	2.0 (40.0%)
Mostly false	4.0 (20.0%)	4.0 (22.2%)	5.0 (25.0%)	2.0 (10.0%)	2.0 (40.0%)	1.0 (20.0%)
Definitely false	10.0 (50.0%)	7.0 (38.9%)	5.0 (25.0%)	1.0 (5.0%)	1.0 (20.0%)	0.0 (0.0%)
missing	0	2	0	0	0	0
**My health is excellent**						
Definitely true	2.0 (10.0%)	3.0 (16.7%)	1.0 (5.0%)	0.0 (0.0%)	0.0 (0.0%)	0.0 (0.0%)
Mostly true	5.0 (25.0%)	12.0 (66.7%)	5.0 (25.0%)	10.0 (50.0%)	1.0 (20.0%)	1.0 (20.0%)
Don’t know	5.0 (25.0%)	2.0 (11.1%)	4.0 (20.0%)	0.0 (0.0%)	0.0 (0.0%)	2.0 (40.0%)
Mostly false	4.0 (20.0%)	1.0 (5.6%)	6.0 (30.0%)	10.0 (50.0%)	2.0 (40.0%)	2.0 (40.0%)
Definitely false	4.0 (20.0%)	0.0 (0.0%)	4.0 (20.0%)	0.0 (0.0%)	2.0 (40.0%)	0.0 (0.0%)
missing	0	2	0	0	0	0

General health visual analogue scores improved in all groups (T1: 90.0 vs 62.5 pre-operatively; T2: 85.0 vs 57.5; T4: 75.0 vs 48.0). SF-36 domains showed consistent favourable with most patients reporting their health as “much better” or “somewhat better” than one year earlier and improvements observed across functional capacity, pain, emotional wellbeing, social functioning and energy levels ( Table 7).

**Table 7.** illustrates EuroQol 5-Dimension 5-Level (EQ-5D-5L) outcomes measured preoperatively, postoperatively and at 1-year follow-up.

	Structured training (T1 & T2)				Conventional Training (T4)	
Characteristics	Pre-surgery (Baseline) n = 20	Post-surgery n = 20	Pre-surgery (Baseline) n = 20	Post-surgery n = 20	Pre-surgery (Baseline) n = 5	Post-surgery n = 5
	T1		T2		T4	
EQ-5D-5L health related Quality of life						
**Mobility, n (%)**						
no problem	3.0 (15.0%)	15.0 (83.3%)	9.0 (45.0%)	14.0 (70.0%)	1.0 (20.0%)	2.0 (40.0%)
slight problem	11.0 (55.0%)	3.0 (16.7%)	1.0 (5.0%)	5.0 (25.0%)	1.0 (20.0%)	2.0 (40.0%)
moderate	4.0 (20.0%)	0.0 (0.0%)	6.0 (30.0%)	1.0 (5.0%)	2.0 (40.0%)	1.0 (20.0%)
severe	2.0 (10.0%)	0.0 (0.0%)	4.0 (20.0%)	0.0 (0.0%)	1.0 (20.0%)	0.0 (0.0%)
Extreme/Unable	0.0 (0.0%)	0.0 (0.0%)	0.0 (0.0%)	0.0 (0.0%)	0.0 (0.0%)	0.0 (0.0%)
missing	0	2	0	0	0	0
**Selfcare, n (%)**						
no problem	5.0 (25.0%)	17.0 (94.4%)	15.0 (75.0%)	19.0 (95.0%)	1.0 (20.0%)	4.0 (80.0%)
slight problem	14.0 (70.0%)	1.0 (5.6%)	2.0 (10.0%)	1.0 (5.0%)	4.0 (80.0%)	1.0 (20.0%)
moderate	1.0 (5.0%)	0.0 (0.0%)	2.0 (10.0%)	0.0 (0.0%)	0.0 (0.0%)	0.0 (0.0%)
severe	0.0 (0.0%)	0.0 (0.0%)	1.0 (5.0%)	0.0 (0.0%)	0.0 (0.0%)	0.0 (0.0%)
Extreme/Unable	0.0 (0.0%)	0.0 (0.0%)	0.0 (0.0%)	0.0 (0.0%)	0.0 (0.0%)	0.0 (0.0%)
missing	0	2	0	0	0	0
**Usual activities**						
Median (Q1, Q3)	1.5 (1.0, 2.0)	0.0 (0.0, 0.0)	1.0 (0.0, 2.0)	0.0 (0.0, 1.0)	2.0 (2.0, 3.0)	0.0 (0.0, 1.0)
Mean (SD)	1.6(0.9)	0.1(0.2)	1.3(1.3)	0.4(0.6)	2.4(1.1)	0.6(0.9)
Missing	0	2				
**Pain, n(%)**						
no problem	2.0 (10.0%)	17.0 (94.4%)	7.0 (35.0%)	14.0 (70.0%)	1.0 (20.0%)	2.0 (40.0%)
slight problem	10.0 (50.0%)	1.0 (5.6%)	4.0 (20.0%)	4.0 (20.0%)	0.0 (0.0%)	3.0 (60.0%)
moderate	6.0 (30.0%)	0.0 (0.0%)	5.0 (25.0%)	1.0 (5.0%)	1.0 (20.0%)	0.0 (0.0%)
severe	2.0 (10.0%)	0.0 (0.0%)	4.0 (20.0%)	1.0 (5.0%)	3.0 (60.0%)	0.0 (0.0%)
Extreme/Unable	0.0 (0.0%)	0.0 (0.0%)	0.0 (0.0%)	0.0 (0.0%)	0.0 (0.0%)	0.0 (0.0%)
Missing	0	2				
**Anxiety**						
no problem	4.0 (20.0%)	15.0 (83.3%)	9.0 (45.0%)	17.0 (85.0%)	1.0 (20.0%)	4.0 (80.0%)
slight problem	10.0 (50.0%)	3.0 (16.7%)	4.0 (20.0%)	3.0 (15.0%)	3.0 (60.0%)	0.0 (0.0%)
moderate	5.0 (25.0%)	0.0 (0.0%)	5.0 (25.0%)	0.0 (0.0%)	1.0 (20.0%)	0.0 (0.0%)
severe	1.0 (5.0%)	0.0 (0.0%)	1.0 (5.0%)	0.0 (0.0%)	0.0 (0.0%)	1.0 (20.0%)
Extreme/Unable	0.0 (0.0%)	0.0 (0.0%)	1.0 (5.0%)	0.0 (0.0%)	0.0 (0.0%)	0.0 (0.0%)
Missing	0	2				
**General health**						
Median (Q1, Q3)	62.5 (50.0, 70.0)	90.0 (85.0, 95.0)	57.5 (42.5, 80.0)	85.0 (75.0, 90.0)	48.0 (20.0, 50.0)	75.0 (60.0, 80.0)
Mean (SD)	61.5(17.8)	89.7(5.4)	62.6(22.4)	82.7(10.4)	40.6(25.9)	72.0(17.5)
Missing	0	2				
**In general, would you say your health is**						
excellent	0.0 (0.0%)	2.0 (11.1%)	3.0 (15.0%)	3.0 (15.0%)	0.0 (0.0%)	0.0 (0.0%)
very good	6.0 (30.0%)	14.0 (77.8%)	3.0 (15.0%)	9.0 (45.0%)	1.0 (20.0%)	3.0 (60.0%)
good	11.0 (55.0%)	2.0 (11.1%)	9.0 (45.0%)	7.0 (35.0%)	0.0 (0.0%)	0.0 (0.0%)
fair	2.0 (10.0%)	0.0 (0.0%)	4.0 (20.0%)	1.0 (5.0%)	3.0 (60.0%)	2.0 (40.0%)
poor	1.0 (5.0%)	0.0 (0.0%)	1.0 (5.0%)	0.0 (0.0%)	1.0 (20.0%)	0.0 (0.0%)
missing	0	2				

## Discussion

Across proximal, random and distal sections, ST group demonstrated consistently better median endothelial integrity (CD34) and lower endothelial stretching. Muscle hypertrophy showed greater variability in CT samples. These findings support the concept that training structure may influence the rate at which proficiency is achieved and consequently may affect conduit preservation during the EVH learning curve.

Structural vascular injury may impair graft performance through platelet aggregation, smooth muscle cell proliferation, neointimal hyperplasia and altered vasoreactivity. ^16^ Although previous studies have assessed structural changes in EVH conduits, evidence during the learning curve remains limited. ^17^ ^,^ ^18^

The VICO trial reported minimal structural damage (n = 2700 sections) but EVH was performed by an experienced practitioner (>500 cases). ^4^ Outcomes during early learning phases, particularly following short, intermittent company-led training, remain poorly characterised. ^2^ ^,^ ^19^

At 6–24 months clinical events were infrequent and the study was not powered to detect differences in MACE and these findings are descriptive only. CT angiography identified flow-limiting and occluded grafts within the ST group. However, affected vessels were classified as ACC/AHA Type C and frequently small-calibre diabetic vessel targets, representing recognised confounders. ^20^ ^,^ ^21^

Although, BAI scores were low, both CT trainees discontinued EVH practice suggesting that self-reported anxiety may not fully capture training-related pressures. ^7^ Future studies should consider incorporating objective stress measures (eg: heart rate variability or cortisol levels) alongside validated questionnaires. ^22^ ^,^ ^23^

### Limitations

This study has several important limitations. Early termination due to the COVID-19 pandemic substantially reduced the planned sample size and small number of trainees and absence of randomisation mean that differences may reflect individual trainee ability or centre-level factors rather than training method alone. Observer variability was not formally quantified. Most of the outcomes reflect those prespecified in the original CREST trial protocol but are reported descriptively. Trainee exposure differed markedly between groups (case volume and continuity), introducing potential performance and experience bias. A predefined competency threshold (≥80%) guided progression in the structured training group. However, formal learning curve modelling and case-number thresholds were not assessed. Incomplete CT angiography follow-up and the observational cluster design without randomisation restrict causal inference. All CT angiography studies were ECG-gated, however functional assessment such as FFR-CT was not performed. Future studies incorporating both anatomical and functional imaging may provide a more comprehensive evaluation of graft performance. CTA interpretation may have been affected by artefacts from surgical clips and adjacent grafts, potentially limiting assessment accuracy in some cases. Additionally, self-reported anxiety (BAI) may underestimate training-related stress, as objective measures were not employed.

## Conclusion

Structured EVH training was associated with more consistent endothelial preservation during the learning curve compared with conventional intermittent training. However, given the small sample size, cluster allocation and absence of randomisation, these findings should be interpreted with caution. No causal inferences can be made, and observed differences may reflect trainee- or centre-level factors. Larger, adequately powered multicentre studies are required to determine whether training structure influences conduit quality and clinically relevant graft outcomes.

### Patient and public involvement and engagement (PPIE)

PPIE was integral to all stages of this study. Patient representatives from the Ticker Club, Manchester Heart Charity, Wythenshawe Hospital were involved from the beginning and helped shape the study design, recruitment approach and participant-facing materials. They reviewed and provided feedback on the study protocol, NIHR postdoc application, participant information sheets, consent forms, data collection materials and ethics application documentation to improve readability, accessibility and relevance to patients undergoing Coronary Artery Bypass Graft (CABG) surgery. Throughout the study, patient voluntary contributors provided advice on study conduct, recruitment of CABG patients, interpretation of findings and dissemination activities. Study findings were communicated through patient and public networks at Manchester Foundation Trust, Ticker club charity newsletters, annual charity educational events, Internationally Society of Minimally Invasive Surgery (ISMICS), Association of Cardiothoracic Surgical Care Practitioner (ACTSCP) conferences to maximise accessibility and impact.

## Data Availability

The dataset is securely stored by the corresponding author (BK) and the study statistician (AR). The datasets generated and analysed during the current study are not publicly available because they contain potentially personal identifiable participant information such as date of birth, gender, ethnicity, place, identification code and were collected under ethical approvals that did not include unrestricted public data sharing. Participants provided consent for the use of their data for the purposes described in the study but not for open public release of individual-level data. Ethical approval for this study was obtained from the Northwest - Greater Manchester South Research Ethics Committee/Institutional Review Board. The approved protocol permits the sharing of de-identified data with researchers upon reasonable request, subject to review of the proposed use and compliance with applicable ethical, legal and data protection requirements. De-identified study data are available from the corresponding author, Professor Bhuvaneswari Krishnamoorthy (BK), at b.bibleraaj@salford.ac.uk, upon reasonable request. Requests should include a brief description of the proposed research purpose and intended use of the data. Access may be granted following review by the study team and where required, evidence of appropriate ethical approval. Data will be shared in accordance with participant consent, institutional policies and applicable data protection regulations. CONSORT checklist is available at: DOI: https://doi.org/10.5281/zenodo.23184054 . Data are available under the terms of the Creative Commons Attribution 4.0 International license (CC-BY 4.0). ^24^
