# Supplementary material for: Structured Vs Conventional Endoscopic Vein Harvesting Training: Vein Integrity and Learning Curve Performance
Source: NIHR Open Res. 2026 Oct 23;6:187. [Version 1] doi: 10.3310/nihropenres.14474.1 (PMC13768713; doi:10.3310/nihropenres.14474.1)
Supplement: Peer review history

# Reviewer report: Reviewer response for version 1

**DOI:** 10.3310/nihropenres.15784.r43156

**Published:** 2026-11-13

**Authors:** Nisha Nair

**Affiliations:** 1 Guy’s and St Thomas’ NHS Foundation Trust, London, UK

Overall Assessment

 This study addresses an important and clinically relevant area in cardiac surgery, exploring the impact of structured Endoscopic Vein Harvesting (EVH) training on vein quality, technical performance and progression towards independent practice. The comparison between structured and conventional training is valuable, particularly as it considers both practical competency and objective assessment of endothelial integrity. However, several methodological limitations, including the very small number of trainees, unequal patient recruitment between groups and premature termination of the original multicentre study, affect the strength and generalisability of the findings. Further clarification is required regarding the training methodology, competency assessment, histological evaluation and clinical outcomes. I would encourage the authors to address the following major and minor comments to strengthen the manuscript and ensure that the conclusions are appropriately supported by the available data.

Is the work clearly and accurately presented and does it cite the current literature?

Yes

If applicable, is the statistical analysis and its interpretation appropriate?

Yes

Are all the source data underlying the results available to ensure full reproducibility?

Partly

Is the study design appropriate and is the work technically sound?

Yes

Are the conclusions drawn adequately supported by the results?

Yes

Are sufficient details of methods and analysis provided to allow replication by others?

Partly

Reviewer Expertise:

Cardiac Surgical Care Practitioner. 24yrs of experience in harvesting conduits for coronary artery bypass grafting.. Via open technique, vein striping and Endoscopic vein harvesting.

I confirm that I have read this submission and believe that I have an appropriate level of expertise to confirm that it is of an acceptable scientific standard.

# Reviewer report: Reviewer response for version 1

**DOI:** 10.3310/nihropenres.15784.r43165

**Published:** 2026-11-11

**Authors:** TANMAY ACHARIA

**Affiliations:** 1 Fortis Hospital, Kolkata, West Bengal, India

This is a good, worthwhile pilot study on an important question: whether structured training protects vein quality during the EVH learning curve. Strengths include blinded histological assessment, a clearly described training programme, prospective registration and candid limitations. The conclusions are appropriately cautious and hypothesis-generating. Minor suggestions: present results per trainee, report why conventionally trained practitioners stopped, add graft-level CT angiography detail, declare the authors' role in developing the programme, and correct small inconsistencies in denominators and table citations. The small, non-randomised design is acknowledged and does not undermine the paper's purpose. Larger multicenter confirmation is the next step.

Is the work clearly and accurately presented and does it cite the current literature?

Yes

If applicable, is the statistical analysis and its interpretation appropriate?

Yes

Are all the source data underlying the results available to ensure full reproducibility?

Yes

Is the study design appropriate and is the work technically sound?

Yes

Are the conclusions drawn adequately supported by the results?

Yes

Are sufficient details of methods and analysis provided to allow replication by others?

Yes

Reviewer Expertise:

N/A

I confirm that I have read this submission and believe that I have an appropriate level of expertise to confirm that it is of an acceptable scientific standard.
